# Cotton *RSG2* Mediates Plant Resistance against *Verticillium dahliae* by miR482b Regulation

**DOI:** 10.3390/biology12070898

**Published:** 2023-06-23

**Authors:** Pan Wu, Chengzhe Lu, Bingting Wang, Feiyan Zhang, Linfang Shi, Yunjiao Xu, Aimin Chen, Huaijun Si, Junji Su, Jiahe Wu

**Affiliations:** 1 State Key Laboratory of Aridland Crop Science, College of Life Science and Technology, Gansu Agricultural University, Lanzhou 730070, China; 2 State Key Laboratory of Plant Genomics, Institute of Microbiology, Chinese Academy of Sciences, Beijing 100101, China; 3 Western Agricultural Research Center, Chinese Academy of Agricultural Sciences, Changji 831100, China; 4 The Key Laboratory for the Creation of Cotton Varieties in the Northwest, Ministry of Agriculture and Rural Affairs, Changji 831100, China

**Keywords:** *Gossypium hirsutum*, *Verticillium dahliae*, ghr-miR482b, GhRSG2

## Abstract

**Simple Summary:**

Cotton is an important economic crop, but its production is constrained by various biotic and abiotic stresses. Verticillium wilt caused by *Verticillium dahliae* is a major factor limiting cotton yield, causing significant losses in both quantity and quality. In plant resistance research, miRNAs are considered important regulatory factors, and the miR482 family is closely related to plant resistance. Typically, this family can target *NBS-LRR* genes to participate in cotton’s defense response to Verticillium wilt, but the specific molecular mechanisms still need further study. This study revealed the mechanism of ghr-miR482b and its target gene *GhRSG2* in cotton’s resistance to Verticillium wilt through molecular biology and biochemistry, providing new ideas and candidate genes for breeding cotton varieties resistant to Verticillium wilt and a reference for the disease-resistant breeding of other crops, thereby improving agricultural productivity, reducing pesticide use, and promoting sustainable agriculture.

**Abstract:**

Cotton Verticillium wilt, mainly caused by *Verticillium dahliae*, has a serious impact on the yield and quality of cotton fiber. Many microRNAs (miRNAs) have been identified to participate in plant resistance to *V*. *dahliae* infection, but the exploration of miRNA’s function mechanism in plant defense is needed. Here, we demonstrate that the ghr-miR482b-*GhRSG2* module mediates cotton plant resistance to *V*. *dahliae* infection. Based on the mRNA degradation data and GUS fusion experiments, ghr-miR482b directedly bonds to *GhRSG2* mRNA to lead to its degradation. The knockdown and overexpression of ghr-miR482b through virus-induced gene silencing strategies enhanced (decreased by 0.39-fold in disease index compared with the control) and weakened (increased by 0.46-fold) the plant resistance to *V*. *dahliae*, respectively. In addition, silencing *GhRSG2* significantly increased (increased by 0.93-fold in disease index) the plant sensitivity to *V*. *dahliae* compared with the control plants treated with empty vector. The expression levels of two SA-related disease genes, *GhPR1* and *GhPR2*, significantly decreased in *GhRSG2*-silenced plants by 0.71 and 0.67 times, respectively, and in ghr-miR482b-overexpressed (OX) plants by 0.59 and 0.75 times, respectively, compared with the control, whereas the expression levels of *GhPR1* and *GhPR2* were significantly increased by 1.21 and 2.59 times, respectively, in ghr-miR482b knockdown (KD) plants. In sum, the ghr-miR482b-*GhRSG2* module participates in the regulation of plant defense against *V*. *dahliae* by inducing the expression of *PR1* and *PR2* genes.

## 1. Introduction

Plant microRNAs (miRNAs) are a type of noncoding small RNA, about 19–24 nucleotides (nt) in length [1]. MiRNA regulates various biological processes, including plant growth and development and defense against abiotic and biotic stresses via post-transcriptional processing of mRNA or translation repression [2,3]. There have been many miRNAs reported to participate in the regulation of plant defense against pathogen infection [4]. For example, *Arabidopsis thaliana* miR393 was reported to target the auxin acceptor TIR1, which disrupted the auxin signal for regulating plant resistance to *Pseudomonas syringae* DC3000 bacteria [5]. Arabidopsis miR393 was also reported to regulate lectin receptor-like kinases associated with lipopolysaccharide (LPS) perception for plant resistance [6]. In cotton, the ghr-miR393-targeted *GhTIR1* affected auxin signal transduction and salicylic acid (SA) synthesis to regulate plant resistance to *Verticillium dahlia* infection [7]. In rice, miR164 targets *NAC60* and regulates rice immunity against the blast fungus *Magnaporthe oryzae* [8]. The cotton miR164-NAC100 module mediates plant resistance to *V. dahliae* at the later stage of infection [9]. Recently, there have been lots of miRNAs reported to participate in plant defense, such as miR773 in Arabidopsis [10], miR168 in Malus [11], milR37 in *Valsa mali* [12], and miR477, miR397, and miR319b in cotton [13,14,15]. For miR482, it was found to be an important miRNA for targeting resistance (*R*) genes to conduct plant disease resistance [16].

miR482 is well known in the miRNA superfamily and was first identified in *Populus trichocarpa* [17]. The miR482 is a 22 nt miRNA and is produced via a normal miRNA biosynthesis pathway [18]. There are more than 20 miR482 members in some plants. For example, there are 24 members in the *Picea abies* genome [19] and 36 members in *G. hirsutum* [20]. The plant miR482 primarily functions in the regulation of post-transcriptional processing of *NBS-LRR* genes, a type of *R* gene essential to plant defense. The miR482 specifically targets the P-loop region of the *NBS-LRR* gene mRNA, which is a conserved sequence motif [21]. miR482 can generally target dozens of *NBS-LRRs* in a single plant species to regulate plant immunity [18].

miR482 has been confirmed to regulate plant resistance to various pathogens in many reports. It is found that this miRNA is generally able to act as a negative regulator on various pathogenic infections in many plant species [21,22]. In tomatoes, the action of miR482 has been deciphered in plant defense. The analysis of tomato miR482-related genetic material, including knockdown, knockout, and overexpression, demonstrated that the miR482-targeted *NBS-LRR* genes’ degradation is associated with secondary siRNA progenesis that regulates plant resistance to several important pathogens, including *Rhizobium*, *Pseudomonas syringae*, *Phytophthora infestans*, *Cucumber mosaic virus*, *Turnip crinkle virus,* and *Tobacco rattle virus* [21,22,23]. The miR482 targeting *NBS-LRR* gene in the regulation of plant resistance to pathogens was also studied in various plant species, such as *Vigna unguiculata* [24], peanut [25], cotton [20,26,27,28], potato [29,30], and soybean [31]. Together, the literature demonstrated that miR482 can target various *NBS-LRR* genes to regulate plant defense against pathogen infection. Therefore, miR482-targeted interaction with *NBS-LRR* gene function in plant resistance to pathogens should be explored in more detail.

The NBS-LRRs in plants are grouped into two major types according to their N-terminal domains: TIR-NBS-LRRs (TNLs) and CC-NBS-LRRs (CNLs) with a Toll-interleukin-like receptor or a coiled-coil motif, respectively [32,33]. The *NBS-LRR R* genes play important roles in plant immune systems as essential components, which participate in two layers of defense: pattern-triggered immunity (PTI) and effector-triggered immunity (ETI) [34,35]. The *NBS-LRR R* genes in both PTI and ETI often activate a series of the same signal pathways, including reactive oxygen species (ROS) burst, MAPK signal cascade, hormone signal pathway, calcium signal, and so on [36]. For example, miR482b-targeted interaction with *NBS-LRR* genes regulated tomato resistance to *Phytophthora infestans* by inhibiting the plant–pathogen interaction pathway, JA and ET signal pathway, and MAPK pathway [37]. The transgenic tomato plants overexpressing sly-miR482e-3p and sly-miR482e-5p decreased the expression levels of *PR1* and *PR5* genes and increased ROS accumulation, but the results were reversed after silencing miR482e-3p and miR482e-5p [38]. Therefore, the novel miR482-targeted *NBS-LRR* genes in plants need to be identified.

Cotton, an important cash crop, provides natural fiber for the textile industry, oil, and protein feedstuffs worldwide [39]. However, cotton production is restricted by various biotic and abiotic stresses. Among these stresses, Verticillium wilt caused by *V. dahliae* is a major factor limiting cotton production, leading to a great loss of fiber yield and quality [40]. Therefore, this study aims to investigate the genes associated with cotton resistance to Verticillium wilt disease, providing candidate genes for the development of resistant cotton cultivars and guiding cotton breeding strategies for disease resistance.

Based on previous studies on the role of miR482 in targeting *NLR* genes for disease resistance [28,30,31], we hypothesize that the cotton miR482b-*RSG2* module participates in the immune response of cotton against *V. dahliae* infection. Here, we characterized a miR482b-*GhRSG2* (a CC-NBS-LRRs (*CNLs*) gene) in plant resistance to *V. dahliae* infection. The ghr-miR482b targets *GhRSG2,* leading to its degradation, according to the analysis of degradome sequencing and the GUS reporter system. The knockdown of ghr-miR482b was able to increase plant resistance to *V. dahliae*, whereas overexpression of ghr-miR482b and silence of *GhRSG2* increased the susceptibility of this fungus. Together, these results showed that ghr-miR482b-*GhRSG2* mediates plant defense and can be used in plant disease-resistant breeding.

## 2. Materials and Methods

### 2.1. Identification of GhCNL Disease Resistance Family

In this study, we obtained the genome data of *G. hirsutum* from the COTTONGEN website (https://www.cottongen.org/, accessed on 19 June 2023) [41]. Coiled-coil motifs were verified in *G. hirsutum* genomes using Paircoil2 (https://cb.csail.mit.edu/cb/paircoil2/, accessed on 19 June 2023), and only those with a P-score lower than 0.02 were retained [42]. To identify NB-ARC and LRR proteins, we employed the HMMER program that utilized the hidden Markov model profile of the NB-ARC domain (PF00931) and LRR domains (PF00560, PF07723, PF07725, PF12799, PF13306, PF13855, and PF14580) in *G. hirsutum* genomes with an E-value < e^−5^ [43]. To confirm the existence of conserved domains, we conducted an additional validation step using the SMART and InterProScan databases [44,45], thereby ensuring a more comprehensive and robust analysis of the dataset.

### 2.2. Plant Materials and Growth Condition

In this study, *G. hirsutum* cv. R15, a plant regeneration line derived from the cultivar “Jihe321”, served as the seed material. Soil culture materials were planted in a nutrient-rich substrate consisting of a 1:1 mixture of nutrient soil and vermiculite, followed by growth in a constant-temperature light incubator under optimal conditions of 28 °C/26 °C (light/dark) with a 16 h light/8 h dark cycle and 65% relative humidity. The hydroponic materials were cultivated using a Hoagland nutrient solution. *Nicotiana benthamiana* plants were grown in vermiculite and maintained in a greenhouse with a 16 h light/8 h dark photoperiod, 65% relative humidity, and irrigated with a B5 nutrient solution [15].

### 2.3. Pathogen Culture, Inoculation, Activation and Disease Detection

To commence the study, a spore suspension of the V991 strain of *V. dahliae* was inoculated on potato dextrose agar (PDA) medium at 28 °C for two days, and subsequently, the mycelium was activated in Czapek’s medium at the same temperature for five days. Following filtration with gauze, the spore concentration was adjusted to 3 × 10^6^ conidia/mL, and plants were inoculated via the root inoculation method as outlined by Gao et al. [46]. Hydroponic seedlings were cultured for three weeks, and samples were obtained at various time points (0, 3, 6, 12, 24, 36, and 48 hpi) to evaluate the relative transcriptional levels of pertinent genes. Additionally, soil-cultured seedlings at the three-leaf stage were infected with the aforementioned spore concentration. After 21 days post-inoculation (dpi), the phenotype of the plants and the brown phenotype of the vascular tissue in the longitudinal section of the stems were observed, and the disease index was determined using the methodology presented by Zhang et al. [47]. The entire set of experiments was conducted in triplicate to ensure the reproducibility of the results.

### 2.4. Fungal Recovery Culture and DNA Abundance Detection

A fungal recovery culture experiment following the protocol by Tang et al. [48] was performed. After 21 days of cotton inoculation, a stem section was collected, treated with 84 disinfectant (the active ingredient is sodium hypochlorite, in which the content of available chlorine is 5.1–5.6%) for 20 min, transferred to a super-clean workstation, washed thrice with sterile water, and a 1 cm stem segment above the cotyledon node was excised and cultured on PDA medium at 28 °C for 5 days. The cultures were preserved after photographing.

After 21 days post-inoculation (dpi), stem DNA was extracted using the CTAB method as described by Wang et al. [49]. The extracted stem DNA was used as a template to detect the abundance of fungal DNA using ITS1-F and ST-Ve1-R as specific primers for *V. dahliae* through RT-qPCR, following the protocol by Xiong et al. [50]. All the above experiments were repeated three times.

### 2.5. RNA Extraction and cDNA Synthesis

Total RNA was extracted from cotton using the miRcute Plant miRNA Isolation Kit (Beijing Tiangen Biotech, Beijing, China). RNA integrity was assessed by 1.2% agarose gel electrophoresis with 1 × TAE electrophoresis buffer at 130 V for 20 min. RNA with a 28S:18S ratio of 2:1 was used for subsequent experiments. The concentration of total RNA was measured by a Nanodrop ND1000 spectrophotometer (NanoDrop Technologies, Wilmington, NC, USA).

The cDNA of the target genes was synthesized using the Easyscript^®^ One-Step gDNA Removal and cDNA Synthesis SuperMix Kit (Beijing TransGen Biotech, Beijing, China). In a 20 µL reaction system, 1 µg total RNA, 10 µL 2 × ES reaction mixture, 1 µL Oligo(dT)_18_ primer, 1 µL Easyscript^®^ RT/RI Enzy Mix, 1 µL gDNA remover, and variable RNase-free water were mixed. The mixture was incubated at 42 °C for 30 min, followed by 85 °C for 5 s. For miRNA cDNA synthesis, a miRNA-specific stem-loop RT primer was used. The reaction was incubated at 16 °C for 30 min, followed by 30 °C for 30 s, 42 °C for 30 s, and 50 °C for 20 s for 60 cycles. Finally, the reaction was stopped at 85 °C for 5 s.

### 2.6. Analysis of Gene Expression by RT-qPCR

Real-time quantitative PCR (RT-qPCR) was performed on a Bio-Rad instrument using the TransStart Top Green qPCR SuperMix Top Green kit (Beijing TransGen Biotech). The reaction mixture (20 µL) consisted of cDNA template (1–2 µL), forward primer (0.4 µL), reverse primer (0.4 µL), 2 × TransStart^®^ Top/Tip Green qPCR SuperMix (10 µL), and variable nuclease-free water. The concentration of all primers was 10 mM. MiRNA utilized *5.8S* as an internal reference gene, while other genes used *GhUBQ7* as an internal reference gene. The method of 2^−ΔΔct^ was used to calculate the relative expression of genes. Three biological replicates were taken from each sample. The data were analyzed using a *t*-test and conformed to both a normal distribution and homogeneity of variance.

### 2.7. Gene Amplification, Vector Construction and Agrobacterium-Mediated Transformation

The precursor miRNA sequence and CDS sequence of genes were amplified from genomic DNA and cDNA, respectively, using primers containing homology arms, restriction sites, and gene-specific sequences. The resulting amplification product was mixed with the corresponding vector, which had been digested with two enzymes and catalyzed by Exnase II (Vazyme Biotech, Nanjing, China) at 37 °C for 30 min. The precursor sequence of ghr-miR482b and the STTM-ghr-miR482b sequence, which contained two incomplete ghr-miR482b mature sequences with three bases (CTA) inserted between the 10th and 11th positions and separated by an artificially designed 48 nt sequence, were inserted into the pTRVe vector to obtain recombinant vectors pTRVe-miR482b OX and pTRVe-miR482b KD, respectively [51]. The best target region of the *GhRSG2* gene was inserted into the pTRV2 vector to obtain the recombinant vector pTRV2-GhRSG2. The precursor sequence of ghr-miR482b was inserted into the pBI121 vector after deletion of the *GUS* gene, while the *GhRSG2* and *mGhRSG2* genes were inserted into the pBI121 vector to obtain recombinant vectors pBI121-miR482b and pBI121-(m) RSG2-GUS, respectively.

The recombinant products were transformed directly into competent cells of *E. coli* DH5α, followed by overnight incubation at 37 °C. Several clones from the transformation plates were selected for colony PCR identification. The positive colonies were used to inoculate liquid Luria Bertani (LB) medium supplemented with kanamycin (50 mg/mL) at 37 °C. The plasmid DNA was extracted using a high-purity plasmid DNA small extraction kit, Plasmid Miniprep Kit (Tsingke Biotech, Beijing, China), and then sent to Tsingke Biotech Company for sequencing.

All correctly sequenced plasmids were transformed into *Agrobacterium tumefaciens* GV3101 by electroporation and stored at −80 °C. A list of all primers used in the experiment is provided in Supplementary Table S1.

### 2.8. Activation, Resuspension and Injection of Agrobacterium

When the cotyledons of cotton seedlings reached complete smoothness, the Agrobacterium solution previously preserved was cultured overnight at 28 °C in liquid Luria Bertani (LB) medium containing kanamycin (50 mg/mL) and rifampicin (50 mg/mL). The Agrobacterium was then resuspended in an MMA solution consisting of 10 mM MgCl_2_, 10 mM 2-morpholinoethanesulfonic acid (MES), and 200 mM acetosyringone (AS), and its OD_600_ value was adjusted to 1.2. For the experiment involving silencing *GhRSG2* genes, pTRV1 (pYL192) was mixed with pTRV2-GhRSG2, pTRV2, and TRV:PDS (phytoene desaturase) in equal volumes, respectively. For ghr-miR482b knockdown and overexpression, pTRV1 (pYL192) was mixed with pTRVe, pTRVe-miR482 OX, pTRVe-miR482 KD, and TRV:PDS in equal volumes, respectively. The mixed resuspension was kept in the dark for 3 h and then injected into the back of smooth cotton cotyledons using a 1 mL syringe. The plants were kept in darkness for 24 h and then transferred to a light incubator. Two weeks later, the leaves of the positive control GhPDS plants were found to be bleached, indicating that the system was working normally.

The chosen bacterial solution was co-injected into the dorsal side of tobacco leaves. Specifically, pBI121-RSG2-GUS was injected in site I, pBI121-mRSG2-GUS in site II, pBI121-miR482b mixed with pBI121-RSG2-GUS in site III, and pBI121-miR482b mixed with pBI121-mRSG2-GUS in site IV.

### 2.9. GUS Histochemical Staining Analysis

Following the injection of Agrobacterium, the tobacco plants were kept in the dark for 24 h and then transferred to a light incubator for further cultivation. After 48 h, samples of tobacco leaves were collected for analysis using GUS histochemical staining.

The GUS histochemical staining analysis of tobacco leaves was performed following the protocol described by Hu et al. [13]. Briefly, tobacco leaves were first immersed in 95% acetone and stored overnight at 4 °C. The leaves were then washed three times with 100 mM PBS buffer (pH 7.0) before being placed in the GUS staining solution. The GUS staining solution was prepared by mixing 1 mM K_3_Fe(CN)_6_, 1 mM K_4_Fe(CN)_6_, 10 mM Na_2_EDTA (pH = 8.0), 0.1% Triton X-100, and 1 mg/mL X-Gluc together. The mixture was filtered in a vacuum for 10 min, and then the leaves were stained overnight at 37 °C to allow the GUS expressed by the transiently transformed *GUS* gene to fully react with the X-Gluc substrate. After the staining reaction was completed, the tobacco leaves were removed and soaked in 75% alcohol for 30 min, then transferred to 95% alcohol for 30 min, and finally soaked in anhydrous ethanol until the green color of the leaves completely faded. The stained tobacco leaves were photographed for preservation.

## 3. Results

### 3.1. Characterization Analysis of Cotton miR482b and CNLs

Our previous report showed that ghr-miR482b is a differentially expressed miRNA between cotton roots inoculated with *V. dahliae* and those treated with mock [9]. The ghr-miR482b is encoded by two genes, *ghr-MIR482b_scaffold3700_A13* and *ghr-MIR482b_D13*, respectively, which generate two different precursors of ghr-miR482b, namely pre-ghr-miR482b-A and pre-ghr-miR482b-D, respectively. They have a typical stem-loop structure where the minimum free energy is −81.90 kcal/mol (A) and −85.50 kcal/mol (B), as predicted by the RNAfold Web Server (Figure S1).

**Figure 1:**
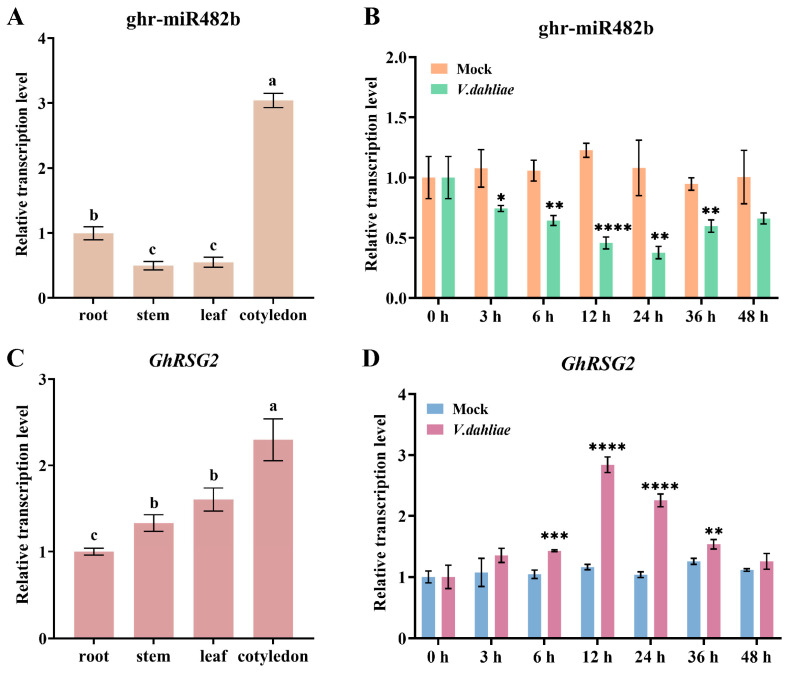
Tissue expression pattern and *V. dahliae*-induced expression pattern of ghr-miR482b and *GhRSG2*. (**A**,**C**) Expression levels of ghr-miR482b and *GhRSG2* in root, stem, leaf, and cotyledon. (**B**,**D**) Expression patterns of ghr-miR482b and *GhRSG2* in mock- and *V. dahliae*-infected leaves at 0, 3, 6, 12, 24, 36 and 48 h. The experiment was conducted in triplicate and analyzed using student’s *t*-test. The values represented the mean ± standard deviation. The different letters indicate significant differences (*p* < 0.05) based on Duncan’s honestly significant differences test. The * represented significant difference, * *p* < 0.05, ** *p* < 0.01, *** *p* < 0.001, **** *p* < 0.0001.

The results of stem-loop qPCR analysis showed that ghr-miR482b constitutively accumulates in cotton root, stem, leaf, and cotyledons, and the highest levels were found in cotyledons (Figure 1A). To confirm the ghr-miR482b response to *V. dahliae* infection, we monitored its expression levels by stem-loop qPCR analysis. It was found that ghr-miR482b accumulation was significantly reduced after 3 h post-inoculation (hpi) compared to the control with mock treatment (Figure 1B). The results demonstrated that ghr-miR482b is able to respond to the *V. dahliae* infection, which indicated that it participates in plant defense.

miR482 generally targets a type of *R* gene, *CNLs*. We identified 83 *GhCNLs* in *G. hirsutum* based on the HMMER, PfamScan, and Paircoil2 programs. There were 26 *GhCNLs* predicted by psRNATarget as ghr-miR482b targets. These GhCNLs contain a P-loop motif targeted by ghr-miR482b. According to our previous degradome sequencing analysis, there were three *GhCNLs* targeted by ghr-miR482b, including Gh_A05G0230, Gh_D05G0314, and Gh_D05G3335 [9]. The three *GhCNLs* responded to *V. dahliae* infection, of which the expression of Gh_D05G3335 was more strongly induced (Figure S2). Gh_D05G3335 was thus selected as the target gene of ghr-miR482b for further research. Gh_D05G3335 was annotated as a *CNL* gene and has a high identity with Arabidopsis *RSG2*, named *GhRSG2*. The qPCR analysis showed that *GhRSG2* was constitutively expressed in the root, stem, leaf, and cotyledon (Figure 1C). Under *V. dahliae* treatment, the *GhRSG2* expression level was significantly increased after 6 hpi, compared to the mock control (Figure 1D), indicating that *GhRSG2* also participates in plant defense.

### 3.2. The GhRSG2 mRNA Degradation Directed by ghr-miR482b

**Figure 2:**
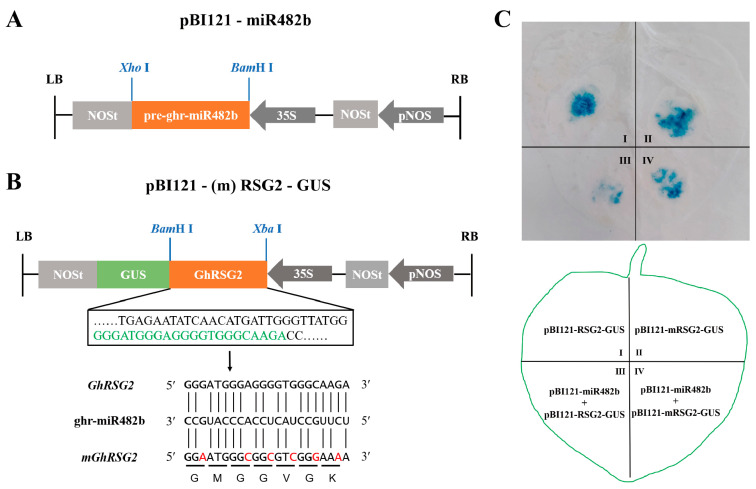
Ghr-miR482b inhibits the expression of *GhRSG2* after transcription. (**A**) Schematic diagram of construction of ghr-miR482b precursor sequence on pBI121 vector. (**B**) Schematic diagram of pBI121-*GhRSG2* and pBI121-RSG2 mutant. The green letter represents the recognition site of ghr-miR482b in *GhRSG2.* Red letters represent mutated bases. (**C**) GUS tissue staining leaf spots (above panel) infiltrated with different vectors as indicated (below panel).

According to the degradome sequencing analysis reported by Hu et al. [9], ghr-miR482b was able to directedly split *GhRSG2* mRNA at 571 nt, corresponding to its 10th and 11th nucleotides (Figure S3). Then, a GUS reporter system was employed to confirm the ghr-miR482-guided function in the degradation of *GhRSG2* mRNA. The pre-ghr-miR482 was driven by the CaMV35S promoter to generate an effector vector (35S::miR482b), and the *GhRSG2* coding sequence and its target sequence mutation, *mGhRSG2*, were fused into *GUS* genes, which were controlled by the CaMV35S promoter to develop two reporter vectors, 35S::*GhRSG2*-GUS and 35S::m*GhRSG2*-GUS, respectively (Figure 2A,B). The leaf spot agroinfiltrated with 35S::*GhRSG2*-GUS or 35S::m*GhRSG2*-GUS alone had normal blue by GUS staining. The leaf spot co-agroinfiltrated with 35S::*GhRSG2*-GUS and 35S::miR482b exhibited a trace blue color, whereas that treated with 35S::m*GhRSG2*-GUS and 35S::miR482b showed a similar blue color to that treated with the reporter vector alone (Figure 2C). Therefore, GUS qualitative analysis showed that ghr-miR482b targets *GhRSG2* to directedly split it through post-transcriptional processing.

### 3.3. The ghr-miR482b Negatively Regulated Plant Resistance to V. dahliae Infection

**Figure 3:**
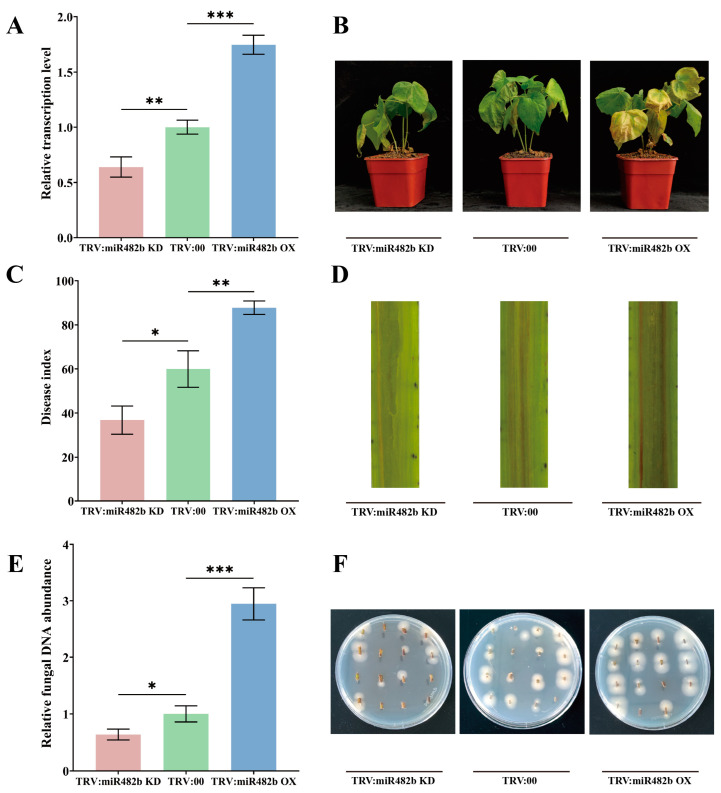
Ghr-miR482b negatively regulates plant resistance to *V. dahliae*. (**A**) Relative expression levels of ghr-miR482b in TRV:miR482b KD and TRV:miR482b OE plants compared with TRV:00 plants. (**B**) Disease phenotypes of TRV:miR482b KD, TRV:miR482b OE and TRV:00 plants. (**C**) Statistics of plant disease index. (**D**) Observation of brown phenotype of xylem in longitudinal section of plant stem. (**E**) Detection of *V. dahliae* DNA relative expression level in stems. (**F**) The growth Phenotype of fungal recovery culture on PDA medium. The average standard deviation came from three repeated experiments. Significant differences in (**A**,**C**,**E**) were determined using Student’s *t*-test (* *p* < 0.05, ** *p* < 0.01, *** *p* < 0.001).

To elucidate the ghr-miR482b function in plant defense, knockdown and overexpression plants were generated by a tobacco rattle virus-induced gene silencing (VIGS) method. The pre-ghr-miR482b coding sequence was inserted in the pTRVe vector under both CaMV35S and sg promoter control to generate an miR482b OX vector (Figure S4A). In addition, the short tandem target mimic (STTM) for ghr-miR482b knockdown (sponge miR482b) was designed under two promoter controls to develop an miR482b KD vector (Figure S4B). The miR482b OX and miR482b KD vectors were transformed into *Agrobacterium* GV3101 by electroporation. Then, cotton seedlings were agroinfiltrated on the cotyledons with *Agrobacterium* containing miR482b OX or miR482b KD vectors. The *PDS*-silencing plants, a marker control, presented albino phenotypes 14 days post-treatment (Figure S5A), while the leaves from these treated plants were sampled to measure ghr-miR482b accumulation by qPCR. As shown in Figure 3A, the miR482b KD plants showed significantly lower ghr-miR482b accumulation than the control plants agroinfiltrated with the empty vector (TRV:00), a reduction of 0.36-fold, whereas the miR482b OX plants accumulated significantly higher ghr-miR482b, an increase of 0.75-fold.

Then, the miR482b OX and miR482b KD plants were inoculated with 10^6^ spores of *V. dahliae* by a root-dipped method to investigate the ghr-miR482b defense function. Twenty-one days post-inoculation (dpi), the control plants showed disease symptoms with yellow wilted leaves, as shown in Figure 3B. In addition, miR482b KD plants showed higher resistance to this fungus compared to the control with fewer yellow wilted leaves, whereas miR482b OX plants exhibited higher susceptibility with more yellow leaves and severe wilting. In connection with these disease symptoms, the disease index (DI) of miR482b KD was significantly lower than the control with a reduction of 0.39-fold, whereas that of miR482b OX was significantly higher with an increase of 0.46-fold (Figure 3C). The longitudinal sections of miR482b OX plant stems showed a deeper color compared to the control, which indicates severe disease symptoms of Verticillium wilt, whereas miR482b KD plant stems exhibited a lighter color (Figure 3D). To verify that ghr-miR482b negatively regulates plant resistance against *V. dahliae* infection, we further monitored the pathogen biomass in infested plants. According to qPCR analysis, fungal content in the miR482b KD plants was significantly lower than the control, whereas that in the miR482b OX plants was significantly higher (Figure 3E). The fungal recovery rate of stem fragments from the miR482b KD plants in media was significantly lower than the control, whereas that from the miR482b OX plants was higher, as shown in Figure 3F. Together, these results showed that ghr-miR482b negatively regulates plant resistance to *V. dahliae* infection.

### 3.4. The Silence of GhRSG2 Reduced Plant Resistance to V. dahliae Infection

**Figure 4:**
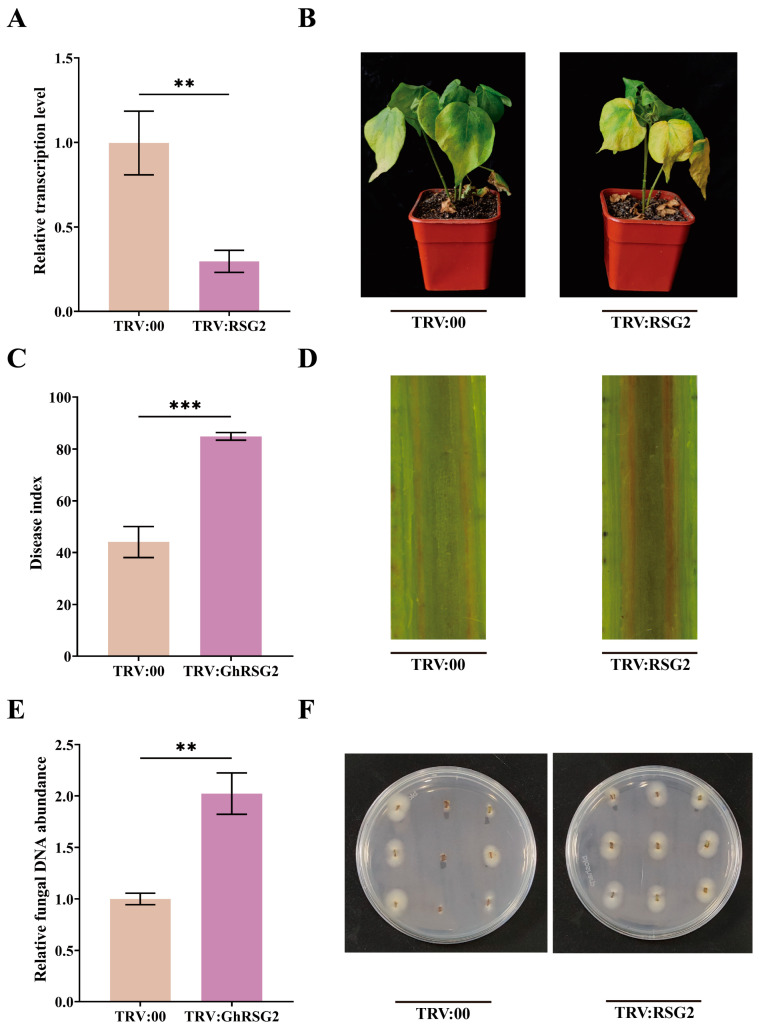
*GhRSG2* gene silencing reduced the resistance of plants to *V. dahliae*. (**A**) Relative transcription level of *GhRSG2* gene in *GhRSG2*-silenced plants. (**B**) Pathogenic phenotypes of TRV:GhRSG2 and TRV:00 plants. (**C**) Disease index of the plants. (**D**) Brown phenotype of vascular tissue. (**E**) DNA detection of *V. dahliae* in stems. (**F**) Observation of fungus recovery culture in stem segments. Means of standard deviations came from three repeated experiments. (**A**,**C**,**E**) by *t*-test, the difference was statistically significant (** *p* < 0.01, *** *p* < 0.001).

To explore the mechanism of ghr-miR482b function in plant resistance, we investigate whether ghr-miR482-targeted *GhRSG2* participates in plant defense. The specific coding sequence of *GhRSG2* was cloned in the pTRV2 vector under the control of the CaMV35S promoter to generate a TRV:GhRSG2 silencing vector, which was transformed into *Agrobacterium* GV3101 by electroporation (Figure S6). The cotton seedlings were agroinfiltrated with this engineered *Agrobacterium* and grown in the greenhouse. Fourteen days post-treatment, the *PDS*-silencing plants presented albino phenotypes (Figure S5B); the *GhRSG2*-silenced plants were sampled to measure the silenced efficiency by qPCR analysis. The results showed that the expression level of *GhRSG2* in silenced plants was significantly reduced by 0.7-fold compared to the control (Figure 4A).

The *GhRSG2*-silenced plants were then inoculated with 10^6^ spores of *V. dahliae*. The silenced plants showed higher susceptibility to pathogen infection with more yellow leaves and severe wilt compared to the control plants at 21 dpi (Figure 4B). Consistent with the results, the DI of *GhRSG2*-silenced plants was higher than the control plants, with an increase of 0.93-fold (Figure 4C). In addition, the longitudinal section of *GhRSG2*-silenced plant stems showed a darker color than that of the control plants, indicating that silenced plants suffered higher damage from the pathogen (Figure 4D). In addition, the fungal content of silenced plants was significantly higher than that of the control plants (Figure 4E). The fungal recovery rate of stem fragments from silenced plants was also higher than that of control plants (Figure 4F). These results demonstrated that *GhRSG2* positively regulates plant resistance against *V. dahliae* infection.

### 3.5. The ghr-miR482b-GhRSG2 Module Mediates Plant Resistance to V. dahliae Infection

**Figure 5:**
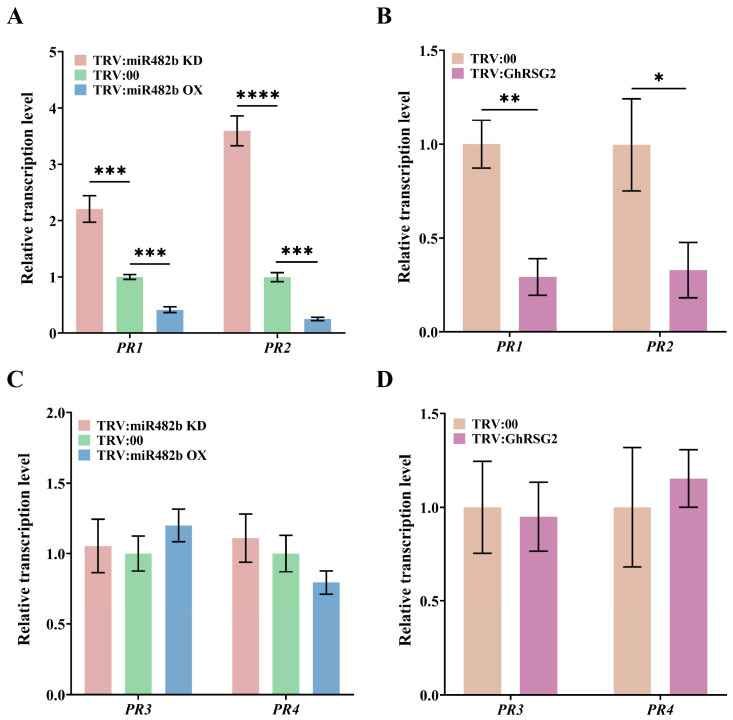
Expression level of defense-related genes in plant leaves after 12 hpi. (**A**,**B**) Relative expression levels of *PR1* and *PR2* genes in leaves of TRV:miR482b KD, TRV:miR482b OE and TRV:GhRSG2 plants after 12 hpi. (**C**,**D**) Relative expression levels of *PR3* and *PR4* genes in leaves were detected by RT-qPCR. *GhUBQ7* is an internal reference gene. The average value of SD was obtained from three repeated experiments. Significant differences were determined by *t*-test (* *p* < 0.05, ** *p* < 0.01, *** *p* < 0.001, **** *p* < 0.0001).

To elucidate the ghr-miR482b-*GhRSG2* module function in plant resistance, we investigated whether the expression levels of defense-related genes in the ghr-miR482b KD, ghr-miR482b OX, and *GhRSG2*-silenced plants changed under *V. dahliae* infection. As shown in Figure 5A,B, compared to the control, SA-related defense genes including *PR1* and *PR2* showed significantly upregulated expression in miR482b KD plants, with increases of 1.21-fold and 2.59-fold, respectively, whereas the expression of *PR1* and *PR2* in miR482b OX and *GhRSG2*-silenced plants showed significantly downregulated expression, with *PR1* expression levels decreased by 0.71-fold and 0.59-fold, and *PR2* expression levels decreased by 0.67-fold and 0.75-fold, respectively. However, JA-related defense genes, including *PR3* and *PR4,* did not show significant differential expression in miR482b KD, miR482b OX, and *GhRSG2*-silenced plants compared to the control plants (Figure 5C,D). These results showed that the ghr-miR482b-*GhRSG2* module mediates plant resistance to *V. dahliae* infection through the expression and promotion of defense-related genes, which are involved in the downstream SA signaling pathway.

## 4. Discussion

Plant miR482 is well known to target *NBS-LRR R* genes and direct their degradation in various species. Notably, there have been increasing reports revealing the function of different miR482 couplings with *NBS-LRR R* genes in plant immunity. In cotton, Zhu et al. reported that cotton miR482 was involved with *GhCNLs*, but the various miR482-*CNLs* modules remain unexplored in plant defense [26,28]. In this study, we found that ghr-miR482b-*GhRSG2* mediates plant resistance to *V. dahliae* infection and is involved in the regulation of *PR* gene induction.

In this study, we observed that ghr-miR482b participates in cotton plant resistance to *V. dahliae* infection. Under *V. dahliae* treatment, ghr-miR482b accumulation was downregulated. The ghr-miR482b overexpression increased plant susceptibility, while ghr-miR482b knockdown increased plant resistance to this fungal pathogen. The result is similar to other reports regarding miR482 function in plant disease resistance. For example, Zhu et al. employed CRISPR/Cas9 to generate various cotton miR482 mutants, which generally negatively regulated plant resistance [28]. The other plant miR482s functioning in defense have been mentioned above, e.g., Arabidopsis, potato, and tomato [23,30,52]. In these miR482-related reports, different target genes were characterized that participate in plant resistance to different pathogens. Therefore, cotton miR482b function dissection confirms the plant miR482 regulation function in plant resistance to *V. dahliae*.

Ghr-miR482b-targeted *GhRSG2* is a typical *NBS-LRR R* gene. The *GhRSG2*-silenced plants showed severe disease symptoms under *V. dahliae* infection like ghr-miR482b OX plants, suggesting that *GhRSG2* positively regulates plant resistance to this fungal pathogen. There are similar reports regarding miR482-targeted *CNLs* in a plant immunity response. For example, miR482-targeted *NBS-LRR* genes have been shown to regulate potato resistance during *V. dahliae* infection [30]. In cotton, some *NBS-LRR* genes targeted by miR482 positively regulate plant resistance to *V. dahliae* infection [26,28]. In this study, *GhRSG2* was verified to be the target of miR482b to regulate cotton plant resistance. Therefore, miR482-targeted *NBS-LRR* genes play important roles in plant defense against pathogen infection.

In this study, it was found that the *PR1* and *PR2* expression levels were higher in miR482b KD plants compared to the control under the fungus infection, whereas the expression levels of these genes in the miR482b OX and *GhRSG2*-silenced plants were lower. These results are consistent with the reports on *NBS-LRR* genes.

It was known that *NBS-LRR* genes belong to one of the largest families of resistance proteins, which can recognize specific pathogens and initiate defense responses at all levels, including upregulated expression of *PR* genes [53,54]. For example, wheat *TaRCR1* increases plant resistance to *Rhizoctonia cerealis* by up-regulating the *PR* gene [55]. Overexpression of peanut *NBS-LRR* genes in tobacco enhances plant resistance to *Ralstonia solanacearum* through up-regulation of the *PR* gene [56]. In sum, the ghr-miR482b-*GhRSG2* module mediates cotton plant defense against *V. dahliae* through regulation of the expression of *PR* genes.

## 5. Conclusions

In this study, we demonstrated that the ghr-miR482b-*GhRSG2* module regulates the resistance of *G. hirsutum* to *V. dahliae* through the induction of *PR1* and *PR2* genes, which could be conserved in plant–fungus interactions and be further explored in the future. The findings provide new insights and candidate genes for breeding Verticillium wilt-resistant cotton cultivars. Further studies can explore the role of the ghr-miR482b-*GhRSG2* module in cotton disease resistance mechanisms and its interactions with other disease-related genes. Moreover, this research could potentially provide references for disease-resistant breeding of other crops, especially those threatened by similar pathogens. A better understanding of the function and regulatory mechanism of the ghr-miR482b-*GhRSG2* module will support the development of more disease-resistant crops, which could improve agricultural productivity, reduce pesticide use, and promote sustainable agricultural development.

## Data Availability

Not applicable.

## Supplementary Materials

The following supporting information can be downloaded at: https://www.mdpi.com/article/10.3390/biology12070898/s1, Figure S1: Secondary structures of two ghr-miR482b precursors located on A (A) and D (B) genomes, respectively. Figure S2: Relative transcription level of three *GhCNLs* genes in mock- and *V. dahliae*-infected leaves at 12 h. Figure S3: Degradation group data of ghr-miR482b directed the cleavage of Gh_D05G3335 transcripts at 571 nt. Figure S4: Schematic diagram of vectors for overexpression and knockdown of ghr-miR482b. Figure S5: Albino phenotype of GhPDS plants 10 days after injection of Agrobacterium. Figure S6: Schematic diagram of *GhRSG2* silent vector. Table S1: Primers used in this study.
