# Supplementary material for: Cotton RSG2 Mediates Plant Resistance against Verticillium dahliae by miR482b Regulation
Source: Biology (Basel). 2023 Jun 23;12(7):898. doi: 10.3390/biology12070898 (PMC10376429; doi:10.3390/biology12070898)
Supplement: Supplementary file 1 [file biology-12-00898-s001.zip › Supplemental Figures.pdf]

Supplementary Figures

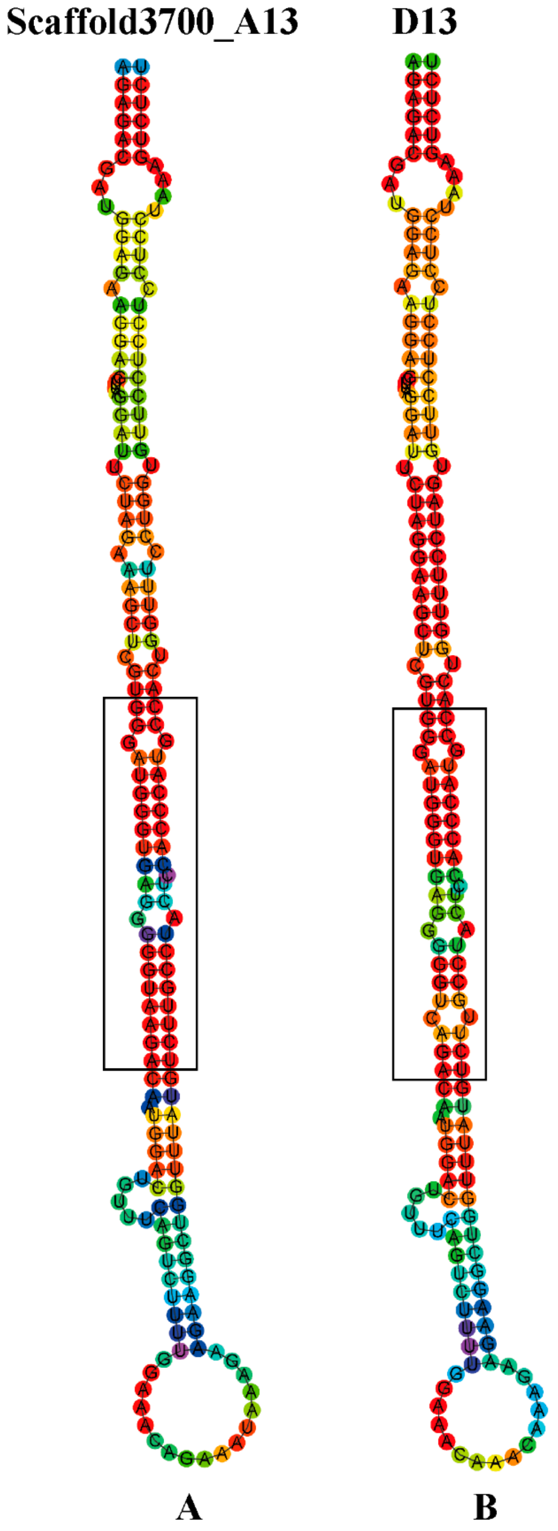

Figure S1. Secondary structures of two ghr-miR482b precursors located on A (A) and D (B) genome, respectively. The black box area represents the location of the mature sequence of ghr-miR482b.

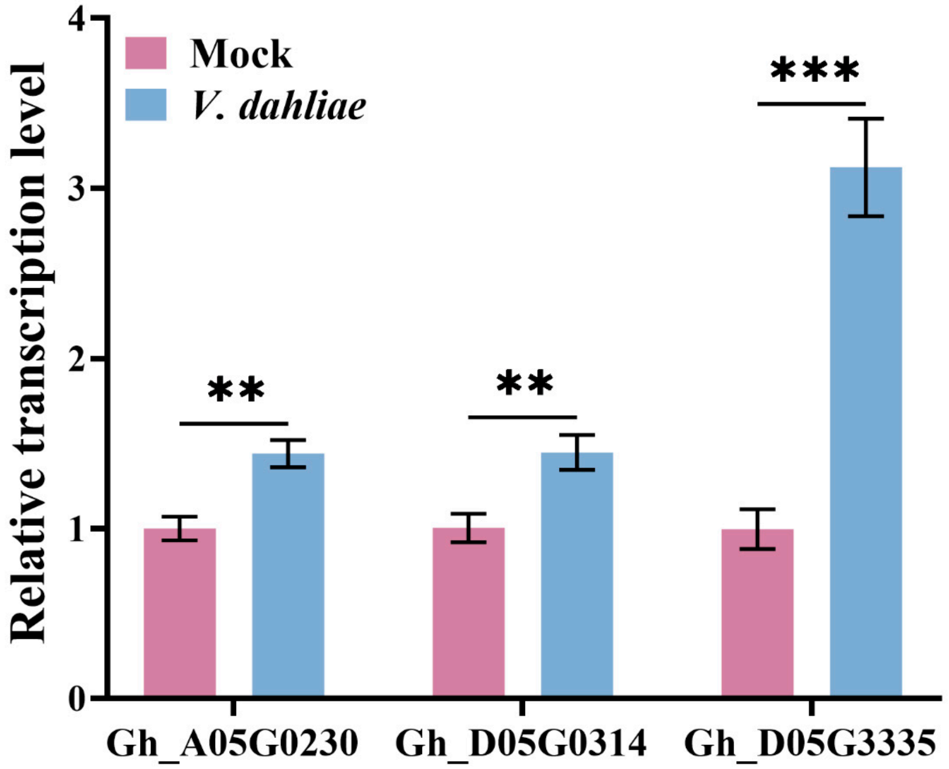

Figure S2. Relative transcription level of three *GhCNLs* genes in mock- and *V. dahliae*-infected leaves at 12 h. The experiment was carried out three times and analyzed using Student's *t*-test. The values represent the mean ± standard deviation. The * represented significant difference, ** $p < 0.01$, *** $p < 0.001$.

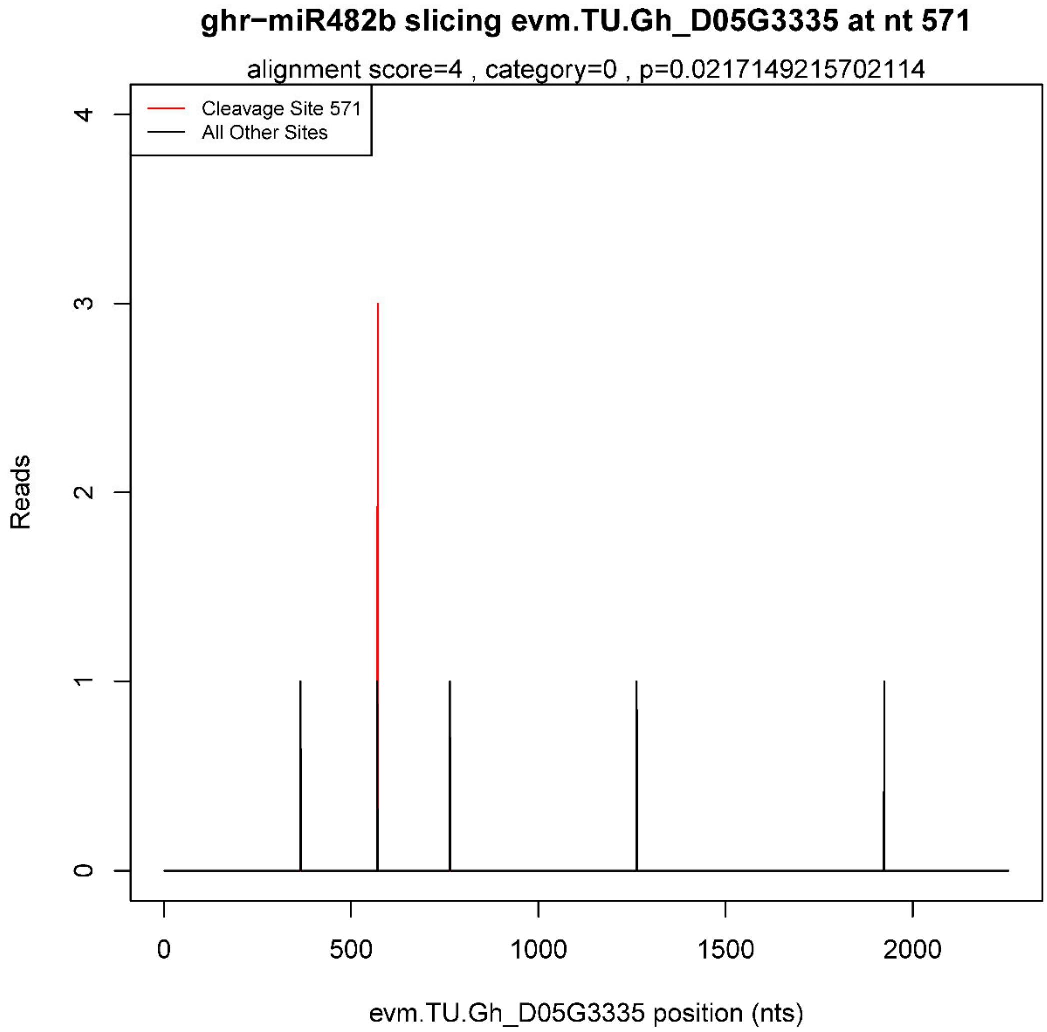

Figure S3. Degradation group data of ghr-miR482b directed the cleavage of Gh_D05G3335 transcripts at 571 nt.

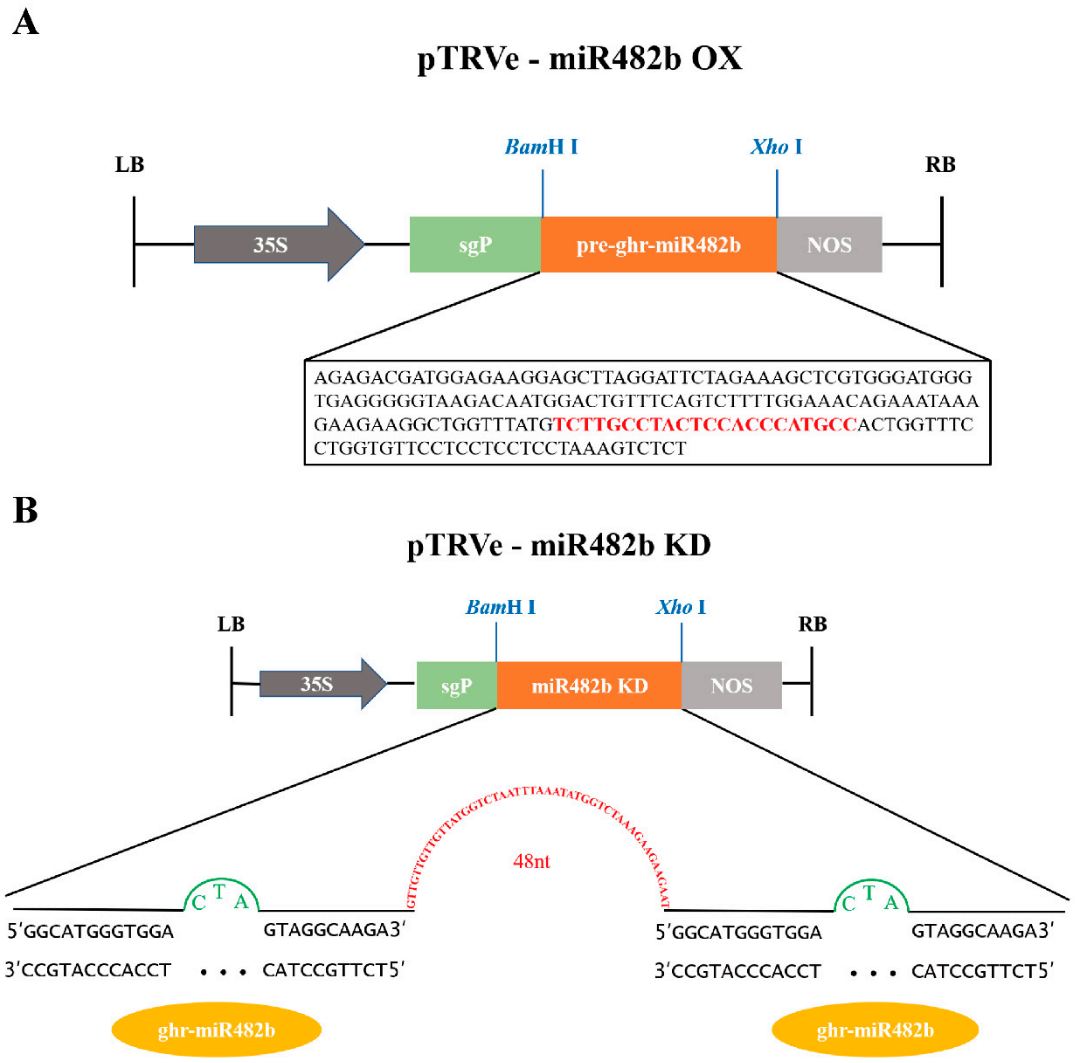

Figure S4. Schematic diagram of vectors for overexpression and knockdown of ghr-miR482b. **(A)** Schematic diagram of the vector for ghr-miR482b overexpression. **(B)** Schematic diagram of the vector for ghr-miR482b knockdown.

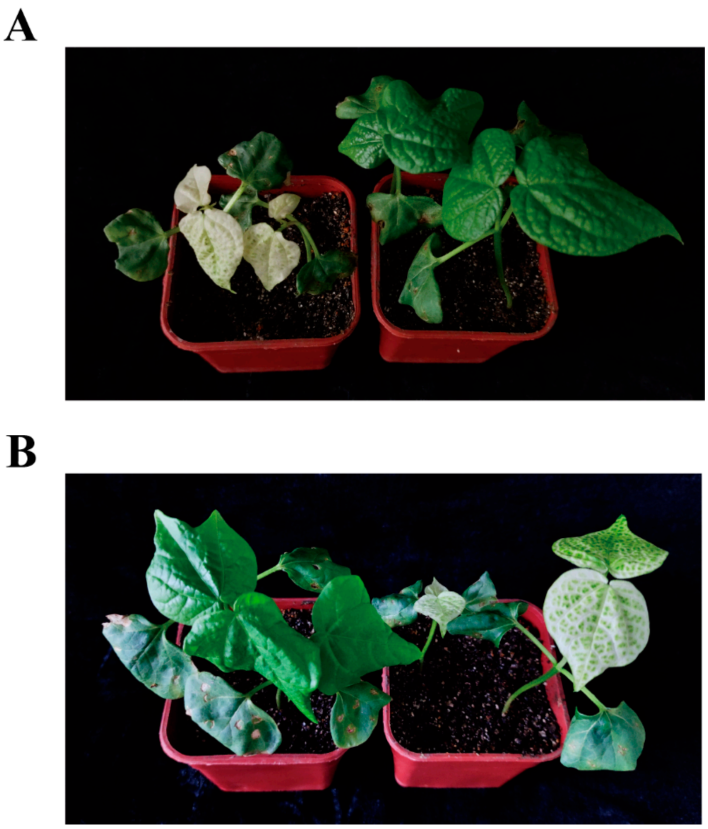

Figure S5. Albino phenotype of GhPDS plants 10 days after injection of Agrobacterium. **(A)** Albino phenotype of GhPDS plants in ghr-miR482b overexpression and knockdown experiments. **(B)** Albino phenotype of GhPDS plants in *GhRSG2* silencing experiments.

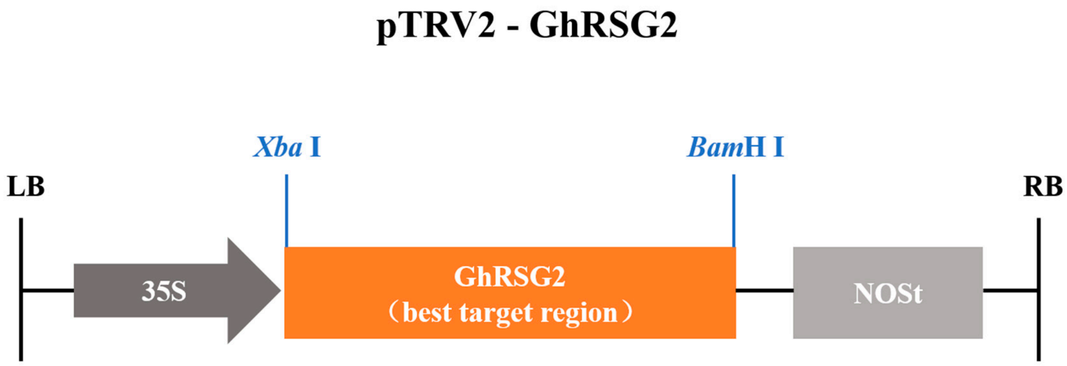

Figure S6. Schematic diagram of *GhRSG2* silent vector.
